# Resolving Discrepancies in Idylla *BRAF* Mutational Assay Results Using Targeted Next-Generation Sequencing

**DOI:** 10.3390/genes15050527

**Published:** 2024-04-23

**Authors:** Giby V. George, Huijie Liu, Audrey N. Jajosky, Zoltán N. Oltvai

**Affiliations:** Department of Pathology and Laboratory Medicine, University of Rochester Medical Center, Rochester, NY 14642, USA

**Keywords:** next-generation sequencing, ultra-rapid PCR

## Abstract

*BRAF* mutation identification is important for the diagnosis and treatment of several tumor types, both solid and hematologic. Rapid identification of *BRAF* mutations is required to determine eligibility for targeted *BRAF* inhibitor therapy. The Idylla *BRAF* mutation assay is a rapid, multiplex allele-specific PCR test designed to detect the most common oncogenic *BRAF* V600 mutations in formalin-fixed paraffin-embedded (FFPE) tissue samples. Here, we describe the validation of the Idylla *BRAF* mutation assay in our laboratory. During routine clinical practice, we noticed cases in which *BRAF* V600 mutations were identified with unusual amplification curves, with three cases displaying a delayed amplification within a double amplification pattern and two false-positive calls. We therefore initiated a quality improvement effort to systematically and retrospectively evaluate next-generation sequencing (NGS)-tested cases with *BRAF* mutations identified within five amino acids of *BRAF* codon V600 and did not identify additional false-positive cases. We hypothesize that late amplification in a double amplification pattern may represent non-specific amplification, whereas cases displaying single delayed amplification curves may stem from the presence of either non-V600 variants, very low-level V600 variants, cytosine deamination artifacts, and/or non-specific amplification by an allele-specific PCR primer. Regardless, we recommend that Idylla *BRAF* cases with non-classical amplification curves undergo reflex NGS testing. These findings are likely relevant for other Idylla assays interrogating hotspot mutations in genes such as *EGFR*, *IDH1*/2, *KRAS*, and *NRAS*.

## 1. Introduction

Detection of *BRAF* mutations is clinically important in several tumor types. For instance, nearly 50% of UV exposure-related melanomas contain *BRAF* mutations, with p.V600E (i.e., a valine to glutamic acid substitution) being the most common (>90% of all *BRAF* mutations) [1,2]. Other solid tumors that commonly harbor *BRAF* V600E mutations include papillary thyroid cancer, serous ovarian cancer, and colorectal cancer [3], to list a few, while *BRAF* V600E-mutant hematologic malignancies include hairy cell leukemia (HCL) [4,5] and histiocytic neoplasms, such as Langerhans cell histiocytosis (LCH) [3]. As such, the rapid identification of *BRAF* mutational status is useful for diagnostic purposes and/or to determine eligibility for FDA-approved targeted *BRAF* inhibitors, such as vemurafenib, dabrafenib, or encorafenib [6,7,8].

Compared to next-generation sequencing (NGS), the turn-around time (TAT) for rapid multiplex polymerase chain reaction (PCR) is a mere 1.5–3 h, enabling faster mutation identification for initiation of targeted therapy. The Idylla *BRAF* mutation assay is a multiplex PCR-based assay [9] designed to detect the most common oncogenic *BRAF* V600 mutations (denoted as V600E/E2/D or V600K/R/M) in purified DNA and formalin-fixed paraffin-embedded (FFPE) specimens. Previous studies have established that a subset of *BRAF* V600-bearing tumor samples contain additional oncogenic or likely oncogenic variants in other genes [10]. However, the specificity of the Idylla *BRAF* assay for detecting *BRAF* V600 variants has not yet been rigorously studied.

We validated the Idylla *BRAF* mutation assay in our laboratory and have performed >200 Idylla *BRAF* assays over the past 30 months. During routine clinical use, we noticed a few cases in which *BRAF* V600 mutations were identified by the Idylla assay but were later found to be discordant with NGS results, including three cases of delayed amplification within a double amplification pattern and two false-positive calls. This prompted us to initiate a quality improvement effort to systematically and retrospectively evaluate cases in which NGS identified *BRAF* mutations within five amino acids upstream or downstream of codon V600 by the Idylla assay. In nine such FFPE specimens identified, the Idylla test resulted in no false-positive calls. We hypothesize that late amplification in a double amplification pattern likely represents non-specific amplification, whereas cases displaying single delayed amplification curves may stem from the presence of either non-V600 variants, very low-level V600 variants, cytosine deamination artifacts in FFPE specimens, and/or non-specific amplification by an allele-specific PCR primer. Regardless, we recommend that Idylla *BRAF* cases with single delayed amplification curves (or even uncertain cases of late amplification with double amplification) undergo reflex NGS testing for further evaluation.

## 2. Materials and Methods

### 2.1. Validation of the Idylla BRAF Mutation Assay

The Idylla *BRAF* mutation assay (Biocartis, Inc., Mechelen, Belgium) is a cartridge-based, automated, rapid multiplex RT-qPCR assay designed to qualitatively detect the V600E/E2/D and V600K/R/M mutations in *BRAF* codon 600 [9]. Automation begins with the FFPE slide(s) and enables nucleic acid extraction, reverse transcription, amplification, and variant detection. However, testing can also be started using previously purified DNA. Each cartridge is intended for one-time use with a single sample. Briefly, the purified DNA or FFPE sample is inserted into the cartridge and the cartridge is placed into the lysis chamber within the Idylla system. Within the lysis chamber, various chemicals and reagents, heat, and high-intensity focused ultrasound enable de-paraffinization and cell lysis. Nucleic acids are purified and concentrated on a solid support and subsequently eluted. After this, they are transferred to the PCR chambers for multiplex RT-qPCR using TaqMan PCR and fluorescence-based detection.

For the purpose of validation, we used previously extracted DNA from clinical samples, CAP proficiency samples, and commercially available reference samples. In total, 14 clinical samples, two commercially available reference samples, and three CAP proficiency samples were tested at 50 ng DNA. All samples had been tested for *BRAF* mutational status via previously validated assays. The reference materials contained *BRAF* mutations at variant allele frequencies (VAFs) confirmed by digital droplet PCR (ddPCR) by the manufacturer.

In addition, 18 clinical formalin-fixed, paraffin-embedded (FFPE) samples were obtained; of these, 12 were known to be positive for *BRAF* V600E/E2/D mutations, while the remaining 6 samples were *BRAF* wild type. Specimens included lymph node, lung, pleural fluid, skin, brain, and colon tissue. In all cases, FFPE tissue (10 μm thick) was directly scraped off slides and sandwiched between two pieces of moistened (with H_2_O) 0.5 cm^2^ Whatman paper and then placed into the cartridge with tweezers. qPCR curves for all reactions were visually observed using the Idylla Explore web-based interface.

The two commercially available samples were serially diluted with Hapmap normal control DNA NA12878 to attain different VAFs. All runs were performed by directly pipetting extracted nucleic acid into the cartridge. Initial validation runs used a minimum of 5 μL input volume. No maximum volume has been established. The assay was performed at the input levels of 12.5 ng, 25 ng, and 50 ng. The commercial samples were tested to determine the lowest permissible input amount.

All samples were tested for assay performance (nucleic acid variant concordance and tissue variant concordance), limit of detection, input limit, precision and reproducibility, and quality control metrics.

### 2.2. Assessment of BRAF Mutational Status in Routine Clinical Samples

In addition to rapid multiplex PCR, orthogonal targeted DNA-based NGS was performed using ThermoFisher’s 35-gene Oncomine Focus Assay (OFA), as previously described [11]. For the purpose of quality assessment, additional cases were identified retrospectively based on NGS results that showed *BRAF* mutations within five amino acids of codon V600. The potential diagnostic and therapeutic significance of the discrepant results were assessed.

## 3. Results

### 3.1. Validation of the Idylla BRAF Mutation Assay

The BioCartis Idylla *BRAF* mutation assay is a rapid testing modality for the qualitative assessment of the most common mutations in codon 600 of the *BRAF* oncogene; it has an impressive TAT of approximately 90 min and a technologist hands-on time of fewer than two minutes. To complement our in-house targeted NGS panels, we validated the Idylla *BRAF* mutation assay using purified DNA from a total of 19 samples, including 14 clinical samples, three CAP proficiency samples, and two commercially available reference samples. All samples had been tested for *BRAF* mutational status via previously validated assays. The reference samples contained *BRAF* mutations at variant allele frequencies (VAFs) confirmed by digital droplet (dd) PCR by the manufacturer. The clinical samples were orthogonally tested using NGS. Using the Idylla *BRAF* assay, across all sample types, 12 samples were identified to have mutations in *BRAF* V600E/E2/D, three possessed mutations in *BRAF* V600K/R/M, and four samples were found to be *BRAF* wild type.

*Assay Performance*: Four of the clinical samples were negative for mutations in *BRAF*, and the remainder of the samples contained one *BRAF* variant covered by the assay. The commercial reference standard HD705 (Horizon Discovery, Shanghai, China) contained *BRAF* c.1799T > A, p.Val600Glu (p.V600E) at a VAF of 5%, and the standard HD123 contained *BRAF* c.1798_1799GtdelinsAA, p.Val600Lys (p.V600K) at a 5% VAF. The Idylla *BRAF* assay produced negative calls or expected variant calls in all cases (Supplementary Table S1).

Additionally, 18 clinical FFPE samples, including 12 *BRAF*-mutation positive and six *BRAF* wild type, were directly tested (i.e., without prior DNA purification) during the validation process (Supplementary Table S2). The Idylla assay was able to identify all variants correctly in all *BRAF*-mutant samples. The Idylla assay also yielded the same negative results as the reference method for the *BRAF* V600 mutation negative samples. Two of the 18 samples were also re-checked with the respective purified DNA form. The result of the direct FFPE sample input was identical to that obtained on the extracted DNA sample. When considering these findings, the clinical sensitivity and specificity of the *BRAF* mutation Idylla assay were 100%.

**Figure 1:**
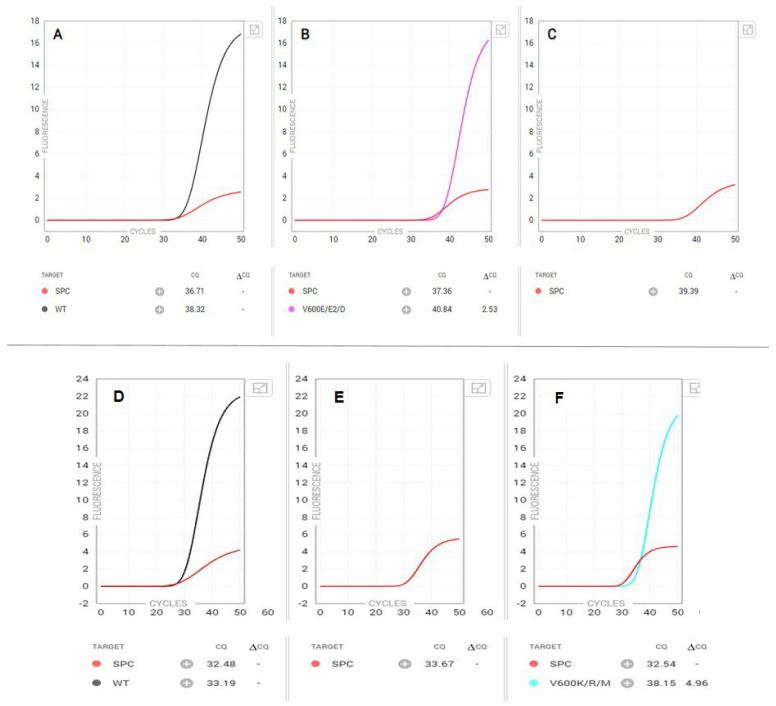
Idylla *BRAF* classic PCR amplification curves for (**top**) *BRAF* V600E/E2/D and (**bottom**) V600K/R/M variants. The top panel displays (**A**) the PCR amplification curve for wild type *BRAF*, (**B**) a positive result for a *BRAF* V600E/E2/D mutation, and (**C**) a negative result for a K/R/M variant. Subsequently performed NGS on this specimen identified a *BRAF* c.1799T > A (p.Val600Glu) variant at a VAF of 53%. The bottom panel displays (**D**) the PCR amplification curve for wild type *BRAF*, (**E**) a negative result for a V600E/E2/D variant, and (**F**) a positive result for a *BRAF* V600K/R/M variant. Confirmatory NGS identified a *BRAF* c.1798_1799delGTinsAG (p.Val600Arg) variant at a VAF of 43%.

A classic *BRAF* V600-positive amplification curve is depicted in Figure 1 (top panel), in which a V600E/E2/D mutation was detected by the Idylla assay. This corresponded to the NGS results, which revealed a *BRAF* c.1799T > A, p.Val600Glu mutation with a VAF of 53%. Figure 1 (bottom panel) highlights another classic case, in which a *BRAF* V600 K/R/M was detected by the Idylla *BRAF* assay. NGS later revealed two *BRAF* mutations: *BRAF* c.1798_1799delGTinsAG, p.Val600Arg (VAF: 43%) and *BRAF* c.910G > A, p.Glu304Lys (VAF: 40%) and a sub-clonal *CTNNB1* c.133T > C, p.Ser45Pro mutation with a VAF of 3%, which was consistent with the Idylla BRAF test result.

*Limit of Detection*: Two commercial standard DNA samples were serially diluted with Hapmap normal control DNA NA12878 to attain different VAFs. The HD705 contained *BRAF* c.1799T > A, p.Val600Glu at a 5% VAF. The HD123 contained *BRAF* c.1798_1799GTdelinsAA, p.Val600Lys at a 5% VAF. Idylla was able to correctly call the *BRAF* V600E and V600K variants at VAFs as low as 1.25% with 50 ng input DNA (Supplementary Table S3). Thus, the limit of detection was determined to be 1.25% VAF and above for *BRAF* V600E and V600K variants at an input of 50 ng DNA.

*Input Limit*: We tested two commercial *BRAF* standard DNA mixtures to determine the lowest permissible input amount. The aforementioned VAFs were noted. The assay was performed at three input levels (12.5 ng, 25 ng, and 50 ng DNA). The *BRAF* V600E and V600K variants were successfully detected at all three input amounts (Supplementary Table S4).

We further established the lowest permissible DNA input for variant detection at 1.25% VAF. The lowest DNA input required for the Idylla *BRAF* cartridge to detect both *BRAF* variants at 1.25% VAF was 50 ng (Supplementary Table S4). Taken together, the required input for the assay is at least 50 ng DNA to ensure variants with the lowest VAFs will be detected. Should a specimen contain a low tumor cellularity percentage, macro-dissection may be performed to enrich the tumor. Lower inputs may be attempted in cases of limited nucleic acid; however, the limit of detection will be compromised. Negative results in such cases should be considered uninformative.

*Precision and Reproducibility*: The assay was performed using three *BRAF*-positive and three *BRAF*-negative clinical samples. We assessed inter-technologist reproducibility by testing each sample on different days by two technologists. Intra-instrument reproducibility was assessed by testing each sample using different instruments on different days. In all cases, results were concordant and the range of ΔCq values between triplicate runs was ≤1.0 cycles (Supplementary Table S5). Thus, the assay was deemed to be highly reproducible.

*Quality Control Metrics*: Metrics for determining sufficient DNA input (valid assay) were established by the manufacturer. When the console displays a “MUTATION DETECTED IN BRAF CODON 600” result, in case of a valid assay, the variant is either *BRAF* V600E/E2/D or V600K/R/M. Both the PCRs for wild type *BRAF* and the V600E/E2/D or V600K/R/M variants will have generated valid curves, indicating that a mutation has been detected in codon 600 of the *BRAF* gene.

Conversely, when the console shows a “NO MUTATION DETECTED” result, only the PCR for wild type *BRAF* has generated a valid curve, and no mutations in *BRAF* V600E/E2/D or V600K/R/M have been detected within the validated ΔCq range. The result is dependent on the integrity of the specimen DNA, the percentage of mutant sequences present in the specimen, the absence of inhibiting substances, and the presence of sufficient amplifiable DNA.

In the case of a limited DNA sample, a *BRAF*-negative sample will be marked with V600K/R/M mutation <5% and may not be detected. Should the console display “INSUFFICIENT DNA INPUT,” it indicates that the DNA amount in the sample is out of range (too low or too high) to ascertain a reliable genotype call. Repeating the assay with a new cartridge, using more sample input but not exceeding the maximum allowed amount of sample, may rectify this issue.

An INVALID result may be reported in the following scenarios: in the presence of inhibitors in the sample, with severe DNA fragmentation potentially caused by a long formalin fixation time, with incorrect placement of a sample in the cartridge, if the sample volume is out of range, if no sample is added, if cartridges are incorrectly stored, with cartridges that have exceeded the in-use period after removal from the pouch, or with cartridge malfunction.

### 3.2. Discrepant Idylla V600-Positive Amplification Profiles and Corresponding NGS Data

Since its validation and implementation in the summer of 2021, our laboratory has successfully performed over 200 Idylla *BRAF* mutational assays on DNA or FFPE slides from various tissues. During the course of routine clinical practice, we also noticed cases in which *BRAF* V600 mutations were identified by the Idylla assay but were later found to be discordant with NGS results. We describe our observations below.

### 3.3. Unusual “Double Amplification” Idylla V600 Amplification Curves and NGS Results

**Figure 2:**
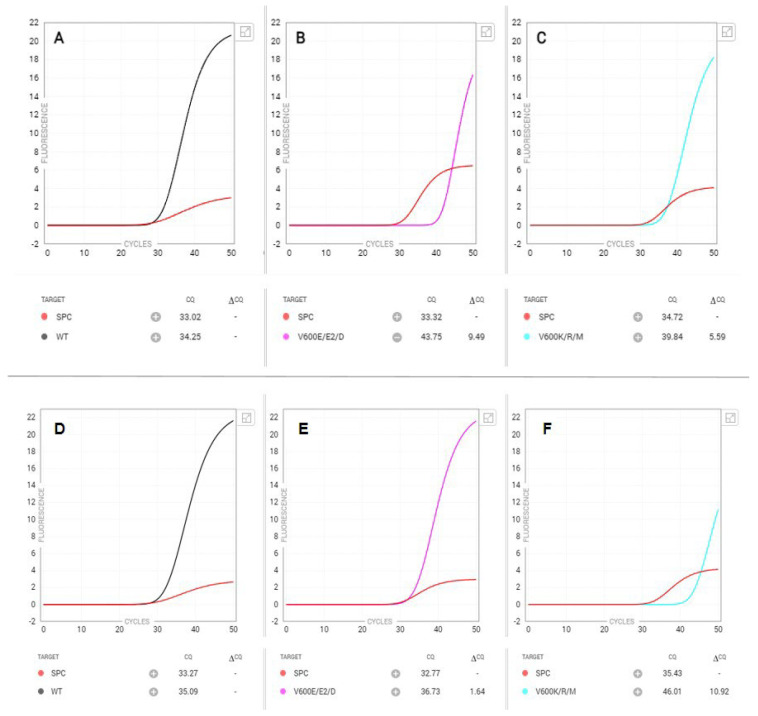
Unusual Idylla BRAF PCR amplification curves. The top panel shows (**A**) the PCR amplification curve for wild type *BRAF*, (**B**) late amplification for a V600E/E2/D variant, and (**C**) early amplification yielding a positive result for a *BRAF* V600K/R/M variant. NGS on the same specimen identified a *BRAF* c.1798_1799delGTinsAA (p.Val600Lys) variant at a VAF of 35%. The bottom panel displays (**D**) the PCR amplification curve for wild type *BRAF*, (**E**) early amplification yielding a positive result for a *BRAF* V600E/E2/D variant, and (**F**) late amplification in the V600K/R/M channel. NGS revealed a *BRAF* c.1799T > A, p.Val600Glu mutation at a VAF of 40% (consistent with the Idylla V600 E/E2/D result) and an additional *MAP2K1* variant.

In one case (Figure 2—top panel), a V600 K/R/M mutation was detected by the Idylla *BRAF* assay; however, late amplification was also noticed within the V600 E/E2/D channel. Subsequent NGS revealed a *BRAF* c.1798_1799delGTinsAA, p.Val600Lys mutation at a VAF of 35%, consistent with the Idylla *BRAF* V600 K/R/M result. Manual inspection of the sequencing reads did not identify an additional *BRAF* V600 variant above the background level.

Similarly, in another case (Figure 2—bottom panel), a *BRAF* V600 E/E2/D mutation was identified by the Idylla *BRAF* assay as depicted by early amplification; however, late amplification was also observed in the *BRAF* V600 K/R/M channel. NGS revealed a *BRAF* c.1799T > A, p.Val600Glu mutation at a VAF of 40% (consistent with the Idylla V600 E/E2/D result) and a small, sub-clonal *MAP2K1* c.371C > T, p.Pro124Leu variant at a VAF of 5%. Again, manual inspection of the sequencing reads did not identify an additional *BRAF* V600 variant above the background level. We also identified an additional case with a *BRAF* c.1799T > A, p.Val600Glu mutation at a VAF of 26% (consistent with the observed Idylla V600 E/E2/D result), with additional mutations detected in *ALK*, *ERBB3*, and *PIK3CA*.

### 3.4. False-Positive Idylla BRAF V600 Results

**Figure 3:**
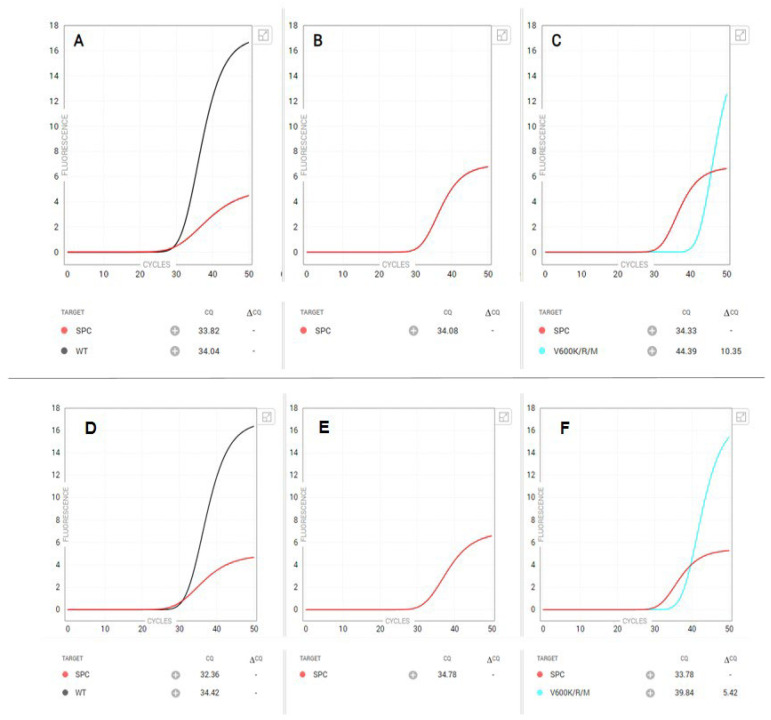
False-positive Idylla *BRAF* PCR amplification curves. The top panel shows (**A**) the PCR amplification curve for wild-type *BRAF*, (**B**) a negative result for a *BRAF* V600E/E2/D variant, and (**C**) a substantially delayed amplification curve in the V600K/R/M channel. NGS on this FFPE specimen identified a *BRAF* c.1789_1790delCTinsTC (p.Leu597Ser) variant at a VAF of 29%, along with mutations in *ALK* and *FGFR3*. The bottom panel displays (**D**) the PCR amplification curve for wild type *BRAF*, (**E**) a negative result for a *BRAF* V600E/E2/D variant, and (**F**) late normal amplification in the V600K/R/M channel. NGS on the same FFPE specimen identified a *BRAF* c.1797_1798insACA (p.Thr599dup) variant at a VAF of 53%.

Additionally, we observed two false-positive calls by the Idylla *BRAF* mutation assay. Figure 3 (top panel) depicts a case with substantially delayed amplification curve in the V600K/R/M channel. NGS on this FFPE specimen identified a *BRAF* c.1789_1790delCTinsTC (p.Leu597Ser) variant at a VAF of 29%, along with mutations in *ALK* and *FGFR3*. Figure 3 (bottom panel) depicts an additional case in which a *BRAF* V600 K/R/M was identified with late normal amplification. NGS on this specimen identified a *BRAF* c.1797_1798insACA (p.Thr599dup) variant at a VAF of 53%.Manual inspection of the sequencing reads did not identify an additional BRAF V600 variant above the background level in either case.

### 3.5. Idylla BRAF Testing of NGS-Detected Non-V600 BRAF Variants

Given the discrepancies and false-positive Idylla results, we initiated an institutional quality improvement effort to systematically and retrospectively evaluate NGS-tested FFPE specimens that were positive for non-V600 *BRAF* variants within five amino acids of codon V600 (n = 9). Of the nine tested specimens with non-V600 *BRAF* variants, none showed any amplification (Figures S1–S9). Thus, only specimens with *BRAF* c.1789_1790delCTinsTC (p.L597S) and c.1797_98insACA (p.T599dup) variants led to false-positive V600 K/R/M amplification curves.

### 3.6. Potential Clinical Significance of Non-V600 BRAF Variants in Idylla BRAF V600-Positive Cases

The Idylla *BRAF* assay is a rapid test requiring minimal technologist hands-on time. However, it remains unclear how frequently *BRAF* V600 mutation-bearing tumors harbor additional *BRAF* variants, which could potentially alter their sensitivity to *BRAF* inhibitor therapy. To address this issue, we reviewed all in-house Idylla *BRAF* V600-positive specimens for which we had corresponding NGS data. As described above, by NGS we identified several samples bearing non-V600 *BRAF* variants, which, as expected, proved negative by the Idylla *BRAF* V600 assay (see Figures S1–S9). However, some of these variants, such as L597R and L597V, are known to be sensitive to BRAF inhibitors [12,13]; thus, their detection would be of both diagnostic and therapeutic importance.

We searched for specimens bearing two *BRAF* variants, V600 and non-V600. Of 311 *BRAF* V600 mutation-positive specimens, we only discovered two that also harbored a second *BRAF* variant. One of these cases proved to be the aforementioned Idylla *BRAF* V600 mutation-positive specimen (Figure 1—bottom panel) harboring *BRAF* c.1798_1799delGTinsAG, p.Val600Arg, and *BRAF* c.910G > A, p.Glu304Lys variants at a VAF of 43% and 40%, respectively. Using our in-house NGS platforms, the amplicon-based Oncomine Focus Assay (OFA) (ThermoFisher, Inc., Waltham, MA, USA) and Illumina TruSight myeloid assay, we could not determine if these two variants were on the same allele (i.e., in cis) or on different alleles (i.e., in trans), although their VAFs were sufficiently close for either possibility to be plausible.

**Figure 4:**
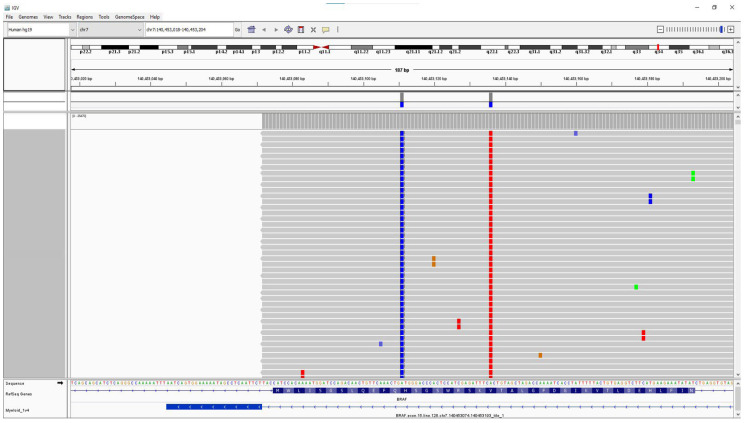
Integrated Genome Viewer (IGV) screenshot. IGV screenshot of a case of hairy cell leukemia (HCL) that proved positive for two *BRAF* variants, c.1799T > A (p.Val600Glu)(red) and c.1824T > G (p.His608Gln)(blue), in cis at VAFs of 14%.

However, we identified one sample from a patient with HCL that was *BRAF* V600 E/E2/D-positive by the Idylla *BRAF* assay, which harbored *BRAF* c.1799T > A (p.Val600Glu) and *BRAF* c.1824T > G (p.His608Gln) variants in cis at VAFs of 14%, as identified by NGS (Figure 4). Of note, both variants are within a region that encodes the protein kinase domain of the B-raf protein. Thus, in a very small subset of cases, both *BRAF* V600 and non-V600 variants can occur on the same allele and may have an influence on the efficacy of *BRAF* V600 mutation-targeted inhibitor therapy.

## 4. Discussion

Identification of *BRAF* mutations is of major importance in the diagnosis of several tumor types, both solid and hematologic. As such, the rapid identification of *BRAF* mutational status is required to determine eligibility for targeted *BRAF* inhibition therapy. In this study, we describe the validation of the Idylla *BRAF* mutation assay and report several cases in which *BRAF* V600 mutations were identified by the Idylla assay but were later found to be discordant with NGS results. We thus initiated an institutional quality improvement effort to resolve these discrepancies.

We validated the Idylla *BRAF* mutation assay using previously extracted DNA from 19 samples, including 14 clinical samples, three CAP proficiency samples, and two commercially available reference samples. All samples had been tested for *BRAF* mutational status via previously validated assays and were tested for assay performance (nucleic acid variant concordance and tissue variant concordance), limit of detection, input limit, precision and reproducibility, and quality control metrics. The clinical samples were orthogonally tested using NGS. Across all sample types, 12 samples were found to have mutations in *BRAF* V600E/E2/D, three possessed mutations in *BRAF* V600K/R/M, and four samples were found to be *BRAF* wild type. Overall, we found the clinical sensitivity and specificity of the *BRAF* mutation Idylla assay to be 100%. Concordant with our validation results, other studies have also shown the Idylla *BRAF* mutation assay to possess high sensitivity, specificity, and remarkable concordance to existing reference methods [9,14].

During the course of routine clinical practice, however, we also noticed two cases in which *BRAF* V600 mutations were identified by the Idylla assay but were later found to be discordant with NGS results (Figure 2). In both cases, we observed double amplification, with early normal amplification in one channel but delayed amplification in a separate channel. NGS, however, only identified a single *BRAF* V600 variant in all three cases. We surmise that late amplification in a double amplification pattern likely represents non-specific amplification and may not necessarily warrant confirmatory NGS testing.

Reciprocally, the presence of non-V600 *BRAF* variants (as identified by NGS) should not lead to an unusual Idylla V600 amplification profile, and even if two *BRAF* variants are present in cis (i.e., affecting the same allele), the Idylla amplification curves should not be affected by the proximity of the second variant. As mentioned prior, we identified a sample from a patient with HCL that was *BRAF* V600 E/E2/D-positive by the Idylla *BRAF* assay and harbored *BRAF* c.1799T > A (p.Val600Glu) and *BRAF* c.1824T > G (p.His608Gln) variants in cis at VAFs of 14%, as identified by NGS (Figure 4). In patients with HCL who develop resistance to first-line therapy with the purine analog 2-chloro-2′-deoxyadenosine [2-CdA] (cladribine), the use of BRAF inhibitor therapy is the standard of care. *BRAF* H608Q has not been biochemically characterized and is considered a variant of unknown significance due to its absence from the gnomAD population database [15]. Of note, the expression of an adjacent variant, *BRAF* Q609H, results in similar cell proliferation and viability levels as wild type B-raf in cell culture [16] and is thus predicted to have no effect on B-raf protein function. Consequently, *BRAF* H608Q may also be a neutral variant in itself. Yet, its presence in cis may affect the binding of allosteric BRAF inhibitors to V600E-mutant BRAF molecules, rendering them less effective, as has been demonstrated prior in a similar double *BRAF* mutation-bearing case [17] and which may be predictable using molecular dynamics simulations [18]. In such rare cases, frequent follow-up testing for minimal residual disease testing by highly sensitive *BRAF* assays may be warranted. 

Additionally, we observed two false-positive calls by the Idylla assay (Figure 3), in which subsequent confirmatory NGS revealed non-V600 *BRAF* variants. This prompted us to initiate an institutional quality improvement effort to evaluate systematically and retrospectively NGS-tested cases within five amino acids of *BRAF* codon V600. Of the nine cases identified, none showed any amplification, confirming the overall high fidelity of the Idylla BRAF assay. We hypothesize that samples with single delayed amplification curves are due to the presence of either non-V600 variants, very low-level V600 variants, cytosine deamination artifacts, and/or non-specific amplification by an allele-specific PCR primer. Regardless, we recommend these cases undergo reflex NGS testing for further evaluation.

Cytosine deamination, in which C/G → T/A, is a possible consequence of histological formalin fixation [19]. Tissue preservation is enabled through formalin cross-linkage with uncharged reactive amino acids; however, rarely, such as in cases of prolonged fixation or exposure to cold ischemia, this preservation process also causes nucleic acid fragmentation [20,21]. In-vivo, this error is repaired by intracellular enzymes, such as uracil DNA glycosylase (UDG)/uracil-N-glycosylase (UNG) and 5-methylcytosine DNA glycosylase [22]. When interpreting NGS data derived from FFPE specimens, low-frequency mutations may reflect such deamination artifacts [19]. We posit whether the two false-positive cases we initially observed may stem from cytosine deamination artifacts consequent to formalin fixation.

Kim et al. report that deamination artifacts may result from poor fixation methods with prolonged exposure, acidic or basic pH, and/or delayed fixation [19]. To mitigate deamination-induced alterations, they implemented UDG pre-treatment, which only affected these changes minimally [19]. Prentice et al. observed no statistical difference in sequencing performance and the number of mutational calls between samples that were pre-treated with UNG versus those that did not undergo pre-treatment until the 48 h fixation mark [21]. After 48 h, they observed a significant increase in deamination artifacts, suggesting that these events may be triggered by a prolonged fixation time [21]. Berra and colleagues support UDG pre-treatment of FFPE specimens intended for NGS, noting a medium reduction in transitions of 80% with sequence artifacts presenting at a VAF < 10% after pre-treatment [22]. Methylated CpG sequences, however, remain despite UDG pre-treatment [19,21].

At our institution, since the fixation times for specimens vary, we are unsure whether this may have caused the false-positive results we observed with the Idylla *BRAF* assay. Interestingly, enhanced DNA integrity has been observed in cold-formalin (4 °C) fixed tissues, which may circumvent the need to monitor formalin fixation times across several specimen types [23]. Although NGS is generally considered the gold standard of mutational analysis, the Idylla *BRAF* mutation assay possesses a number of advantages. Unlike NGS, the Idylla *BRAF* mutation assay is a rapid multiplex RT-qPCR with an impressive TAT of approximately 90 min and a technologist hands-on time of fewer than three minutes. In the case of NGS, low sample volume and the specifics of testing typically require sample batching, with prolonged turnaround times (12–15 days). Unlike the Idylla assay, NGS is also highly complex (with a complicated bioinformatics pipeline) and labor-intensive, requiring highly skilled personnel, precluding its wide use and availability at smaller hospitals. Thus, the Idylla assay serves as an apt, cost-effective alternative for prompt *BRAF* mutation identification to enable targeted therapy.

Our study has several limitations. Our sample size was too limited to achieve general conclusions and our observations were descriptive. Additionally, we were not able to control for variables, such as fixation times, which may affect Idylla and NGS assays differentially. Regardless, we recommend that Idylla *BRAF* cases with single delayed amplification curves (or even uncertain cases of late amplification with double amplification) undergo reflex NGS testing for further evaluation. More generally, given their potential diagnostic and therapeutic significance, Idylla amplification curves of all *BRAF* variants should be inspected and carefully assessed prior to result reporting.

## Data Availability

No new data were created or analyzed in this study. Data sharing is not applicable to this article.

## Supplementary Materials

The following supporting information can be downloaded at: https://www.mdpi.com/article/10.3390/genes15050527/s1. Figures S1–S9: Idylla amplification profiles of NGS-tested specimens with non-V600 BRAF variants that were ±5 amino acids of the BRAF V600 codon. Table S1: Nucleic Acid Variant Concordance; Table S2: Tissue Variant Concordance; Table S3: Limit of Detection; Table S4: Input Limitation for 1.25% and 5% VAF; Table S5: Reproducibility.
