# Supplementary material for: Resolving Discrepancies in Idylla BRAF Mutational Assay Results Using Targeted Next-Generation Sequencing
Source: Genes (Basel). 2024 Apr 23;15(5):527. doi: 10.3390/genes15050527 (PMC11121162; doi:10.3390/genes15050527)

SUPPLEMENTAL MATERIAL

Resolving Discrepancies in Idylla *BRAF* Mutational Assay Results Using Targeted Next-Generation Sequencing

Giby V. George[1], Huijie Liu[1], Audrey N. Jajosky[1], Zoltán N. Oltvai[1]

[1]Department of Pathology and Laboratory Medicine, University of Rochester, Rochester, NY 14627, USA

SUPPLEMENTAL TABLES

Supplemental Table S1 Nucleic Acid Variant Concordance

Sample ID	Result (Idylla)	ΔCq	SPC1 (Cq)	SPC2 (Cq)	SPC3 (Cq)	WT (Cq)	Reference Results	Ref. Meth.	Sample Type
1	BRAF V600E/V600E2/V600D, c.1799T>A/c.1799_1800TG>AA /c.1799_1800TG>AT/ c.1799_1800TG> AC	1.64	37.5	38.2	39.6	38.9	BRAF c.1799T>A, p.Val600Glu	Send-out NGS	FFPE Lung
2	BRAF V600E/V600E2/V600D, c.1799T>A/c.1799_1800TG>AA /c.1799_1800TG>AT/ c.1799_1800TG> AC	0.74	34.6	34.9	34.3	35.2	BRAF c.1799T>A, p.Val600Glu	Send-out NGS	FFPE Lung
3	BRAF V600E/V600E2/V600D, c.1799T>A/c.1799_1800TG>AA /c.1799_1800TG>AT/ c.1799_1800TG> AC	3.67	32.1	32.5	32.9	31.7	BRAF c.1799T>A, p.Val600Glu, VAF 8.72	NGS	FFPE Lung
4	BRAF V600E/V600E2/V600D, c.1799T>A/c.1799_1800TG>AA /c.1799_1800TG>AT/ c.1799_1800TG> AC	3.01	35.6	35.3	36.8	35.9	BRAF c.1799T>A, p.Val600Glu	In house BRAF	FFPE Colon
5	BRAF V600E/V600E2/V600D, c.1799T>A/c.1799_1800TG>AA /c.1799_1800TG>AT/ c.1799_1800TG> AC	3.10	33.4	33	34.9	33.6	BRAF c.1799T>A, p.Val600Glu	In house BRAF	FFPE Colon
6	BRAF V600E/V600E2/V600D, c.1799T>A/c.1799_1800TG>AA /c.1799_1800TG>AT/ c.1799_1800TG> AC	4.65	36.6	36.7	37.8	37.3	BRAF c.1799T>A, p.Val600Glu	Send-out NGS	FFPE Pleura
7*	BRAF V600E/V600E2/V600D, c.1799T>A/c.1799_1800TG>AA /c.1799_1800TG>AT/ c.1799_1800TG> AC	2.86	32.9	32.9	33.9	32.9	BRAF c.1799T>A, p.Val600Glu, VAF 11.94	NGS	FFPE Lung
8	BRAF V600E/V600E2/V600D, c.1799T>A/c.1799_1800TG>AA /c.1799_1800TG>AT/ c.1799_1800TG> AC	2.23	32.6	30.9	30.9	32.5	BRAF c.1799T>A, p.Val600Glu	In house BRAF	FFPE Lung
9	BRAF V600E/V600E2/V600D, c.1799T>A/c.1799_1800TG>AA /c.1799_1800TG>AT/ c.1799_1800TG> AC	0.65	34	34.4	36.6	36.2	BRAF c.1799T>A, p.Val600Glu	NGS	FFPE Lymph node
10	BRAF V600E/V600E2/V600D, c.1799T>A/c.1799_1800TG>AA /c.1799_1800TG>AT/ c.1799_1800TG> AC	1.41	29.9	29.7	31.4	30.3	BRAF c.1799T>A, p.Val600Glu	In house BRAF	FFPE Colon

11	BRAF V600E/V600E2/V600D, c.1799T>A/c.1799_1800TG>AA /c.1799_1800TG>AT/ c.1799_1800TG> AC	1.96	31.1	31	32.8	31.8	BRAF c.1799T>A, p.Val600Glu	In house BRAF	FFPE Colon
12	BRAF V600E/V600E2/V600D, c.1799T>A/c.1799_1800TG>AA /c.1799_1800TG>AT/ c.1799_1800TG> AC	3.35	35.5	34.9	35.9	36	BRAF c.1799T>A, p.Val600Glu	In house BRAF	FFPE Skin
13	No Mutation detected in BRAF Codon 600		32.9	33.6	33.9	32.1	No BRAF Mutation detected	Send-out NGS	FFPE Pleural fluid
14	No Mutation detected in BRAF Codon 600		31.5	31.5	32.7	31.4	No BRAF Mutation detected	Send-out NGS	FFPE Lymph node
15	No Mutation detected in BRAF Codon 600		30.1	30.2	30.9	29.8	No BRAF Mutation detected	Send-out NGS	FFPE Lung
16	No Mutation detected in BRAF Codon 600		31	31.4	30.6	30.3	No BRAF Mutation detected	Send-out NGS	FFPE Brain
17	No Mutation detected in BRAF Codon 600		28.4	27.5	29.9	27	No BRAF Mutation detected	Send-out NGS	FFPE Metastatic lung tumor
18	No Mutation detected in BRAF Codon 600		29.8	29.6	29.6	29.6	No BRAF Mutation detected	Send-out NGS	FFPE Lung

Supplemental Table S2 Tissue Variant Concordance

Sample ID	Result (Idylla)	ΔCq	SPC1 (Cq)	SPC2 (Cq)	SPC3 (Cq)	WT (Cq)	Reference Results	Ref. Meth.	Sample Type
1	BRAF V600E/V600E2/V600D, c.1799T>A/c.1799_1800TG>AA/ c.1799_1800TG>AT/ c.1799_1800TG> AC	1.64	37.5	38.2	39.6	38.9	BRAF c.1799T>A, p.Val600Glu	Send-out NGS	FFPE Lung
2	BRAF V600E/V600E2/V600D, c.1799T>A/c.1799_1800TG>AA/ c.1799_1800TG>AT/ c.1799_1800TG> AC	0.74	34.6	34.9	34.3	35.2	BRAF c.1799T>A, p.Val600Glu	Send-out NGS	FFPE Lung
3	BRAF V600E/V600E2/V600D, c.1799T>A/c.1799_1800TG>AA/ c.1799_1800TG>AT/ c.1799_1800TG> AC	3.67	32.1	32.5	32.9	31.7	BRAF c.1799T>A, p.Val600Glu, VAF 8.72	NGS	FFPE Lung

4	BRAF V600E/V600E2/V600D, c.1799T>A/c.1799_1800TG>AA/ c.1799_1800TG>AT/ c.1799_1800TG> AC	3.01	35.6	35.3	36.8	35.9	BRAF c.1799T>A, p.Val600Glu	In house BRAF	FFPE Colon
5	BRAF V600E/V600E2/V600D, c.1799T>A/c.1799_1800TG>AA/ c.1799_1800TG>AT/ c.1799_1800TG> AC	3.10	33.4	33	34.9	33.6	BRAF c.1799T>A, p.Val600Glu	In house BRAF	FFPE Colon
6	BRAF V600E/V600E2/V600D, c.1799T>A/c.1799_1800TG>AA/ c.1799_1800TG>AT/ c.1799_1800TG> AC	4.65	36.6	36.7	37.8	37.3	BRAF c.1799T>A, p.Val600Glu	Send-out NGS	FFPE Pleura
7*	BRAF V600E/V600E2/V600D, c.1799T>A/c.1799_1800TG>AA/ c.1799_1800TG>AT/ c.1799_1800TG> AC	2.86	32.9	32.9	33.9	32.9	BRAF c.1799T>A, p.Val600Glu, VAF 11.94	NGS	FFPE Lung
8	BRAF V600E/V600E2/V600D, c.1799T>A/c.1799_1800TG>AA/ c.1799_1800TG>AT/ c.1799_1800TG> AC	2.23	32.6	30.9	30.9	32.5	BRAF c.1799T>A, p.Val600Glu	In house BRAF	FFPE Lung
9	BRAF V600E/V600E2/V600D, c.1799T>A/c.1799_1800TG>AA/ c.1799_1800TG>AT/ c.1799_1800TG> AC	0.65	34	34.4	36.6	36.2	BRAF c.1799T>A, p.Val600Glu	NGS	FFPE Lymph node
10	BRAF V600E/V600E2/V600D, c.1799T>A/c.1799_1800TG>AA/ c.1799_1800TG>AT/ c.1799_1800TG> AC	1.41	29.9	29.7	31.4	30.3	BRAF c.1799T>A, p.Val600Glu	In house BRAF	FFPE Colon
11	BRAF V600E/V600E2/V600D, c.1799T>A/c.1799_1800TG>AA/ c.1799_1800TG>AT/ c.1799_1800TG> AC	1.96	31.1	31	32.8	31.8	BRAF c.1799T>A, p.Val600Glu	In house BRAF	FFPE Colon
12	BRAF V600E/V600E2/V600D, c.1799T>A/c.1799_1800TG>AA/ c.1799_1800TG>AT/ c.1799_1800TG> AC	3.35	35.5	34.9	35.9	36	BRAF c.1799T>A, p.Val600Glu	In house BRAF	FFPE Skin
13	No Mutation detected in BRAF Codon 600		32.9	33.6	33.9	32.1	No BRAF Mutation detected	Send-out NGS	FFPE Pleural fluid
14	No Mutation detected in BRAF Codon 600		31.5	31.5	32.7	31.4	No BRAF Mutation detected	Send-out NGS	FFPE Lymph node
15	No Mutation detected in BRAF Codon 600		30.1	30.2	30.9	29.8	No BRAF Mutation detected	Send-out NGS	FFPE Lung
16	No Mutation detected in BRAF Codon 600		31	31.4	30.6	30.3	No BRAF Mutation detected	Send-out NGS	FFPE Brain

17	No Mutation detected in BRAF Codon 600		28.4	27.5	29.9	27	No BRAF Mutation detected	Send-out NGS	FFPE Metastatic lung tumor
18	No Mutation detected in BRAF Codon 600		29.8	29.6	29.6	29.6	No BRAF Mutation detected	Send-out NGS	FFPE Lung

Supplemental Table S3 Limit of Detection

	Dilution	Expected VAF (%)	SPC1 (ΔCq)	SPC2 (ΔCq)	SPC3 (ΔCq)	WT (ΔCq)	ΔCq	Idylla Result
HD705 (BRAF c.1799T>A, p.Val600Glu)	N/A	5	33.7	33.7	35.6	34.4	4.3	Detected
	1:2	2.5	34.4	34.1	35.7	35.1	5.0	Detected
	1:4	1.25	34.2	34.5	36.2	34.8	7.6	Detected
	1:8	0.625	34.4	34.5	34.3	34.9		Not Detected
HD123 (BRAF c.1798_1799GTdelinsAA, p.Val600Lys)	N/A	5	34.3	34.5	35.1	34.8	7.6	Detected
	1:2	2.5	33.8	34.0	35.6	34.6	10.3	Detected
	1:4	1.25	34.1	34.6	34.2	34.5	9.1	Detected
	1:8	0.625	34.4	34.5	34.3	34.9		Not Detected

Supplemental Table S4.1 Input Limitation for 5% VAF

HD705 (BRAF c.1799T>A, p.Val600Glu, VAF 5%)							HD123 (BRAF c.1798_1799GTdelinsAA, p.Val600Lys, VAF 5%)						
Input (ng)	SPC1 (Cq)	SPC2 (Cq)	SPC3 (Cq)	WT (Cq)	ΔCq	Idylla Result	Input (ng)	SPC1 (Cq)	SPC2 (Cq)	SPC3 (Cq)	WT (Cq)	ΔCq	Idylla Result
50	33.7	33.7	35.6	34.4	4.3	Detected	50	34.3	34.5	35.1	34.8	7.6	Detected
25	35.6	36.1	37.0	35.9	5.2	Detected	25	34.8	35.5	35.6	35.6	7.3	Detected
12.5	36.6	37.1	36.7	37.8	5.6	Detected	12.5	35.5	36.2	36.7	36.4	6.9	Detected

Supplemental Table S4.2 Input Limitation for 1.25% VAF

HD705 (BRAF c.1799T>A, p.Val600Glu, VAF 1.25%)							HD123 (BRAF c.1798_1799GTdelinsAA, p.Val600Lys, VAF 1.25%)						
Input (ng)	SPC1 (Cq)	SPC2 (Cq)	SPC3 (Cq)	WT (Cq)	ΔCq	Idylla Result	Input (ng)	SPC1 (Cq)	SPC2 (Cq)	SPC3 (Cq)	WT (Cq)	ΔCq	Idylla Result
100	33.7	33.4	34.5	34.3	5.6	Detected	100	33.2	34.5	34.6	33.8	11.1	Detected
50	34.2	34.5	36.2	34.2	7.6	Detected	50	34.1	34.6	34.2	34.5	9.1	Detected
25	35.9	36.1	36.8	36.8	5.1	Detected	25	35.6	37.3	36.2	36.6		Insuffient

Supplemental Table S5 Reproducibility

Specimen No.	Variant	ΔCq	SPC1 (Cq)	SPC2 (Cq)	SPC1 (Cq)	WT (Cq)
3-1	BRAF V600E/V600E2/V600D, c.1799T>A/c.1799_1800TG>AA/c.1799_1800TG>AT/ c.1799_1800TG>AC	3.7	32.1	32.5	32.9	31.7
3-2	BRAF V600E/V600E2/V600D, c.1799T>A/c.1799_1800TG>AA/c.1799_1800TG>AT/ c.1799_1800TG>AC	4.1	30.9	31.6	31	31.2
3-3	BRAF V600E/V600E2/V600D, c.1799T>A/c.1799_1800TG>AA/c.1799_1800TG>AT/ c.1799_1800TG>AC	3.3	31	30.4	32.4	31
7-1	BRAF V600E/V600E2/V600D, c.1799T>A/c.1799_1800TG>AA/c.1799_1800TG>AT/ c.1799_1800TG>AC	2.9	32.9	32.9	33.9	32.9
7-2	BRAF V600E/V600E2/V600D, c.1799T>A/c.1799_1800TG>AA/c.1799_1800TG>AT/ c.1799_1800TG>AC	3.2	32.9	33.3	34.8	33.2
7-3	BRAF V600E/V600E2/V600D, c.1799T>A/c.1799_1800TG>AA/c.1799_1800TG>AT/ c.1799_1800TG>AC	2.7	33.6	33.2	34.9	33.1
5-1	BRAF V600E/V600E2/V600D, c.1799T>A/c.1799_1800TG>AA/c.1799_1800TG>AT/ c.1799_1800TG>AC	3.1	33.4	33	34.9	33.6
5-2	BRAF V600E/V600E2/V600D, c.1799T>A/c.1799_1800TG>AA/c.1799_1800TG>AT/ c.1799_1800TG>AC	4.1	33	32.5	34.4	33
5-3	BRAF V600E/V600E2/V600D, c.1799T>A/c.1799_1800TG>AA/c.1799_1800TG>AT/ c.1799_1800TG>AC	3.6	33.6	32.7	32.9	34
14-1	No BRAF mutation detected		31.5	31.5	32.7	31.4
14-2	No BRAF mutation detected		30.8	32.3	30.5	30.9
14-3	No BRAF mutation detected		31.4	31.2	33	31.7
15-1	No BRAF mutation detected		30.1	30.2	30.9	29.8
15-2	No BRAF mutation detected		30.6	30.5	30.3	30.2
15-3	No BRAF mutation detected		30.7	30.6	31.1	30.5
18-1	No BRAF mutation detected		29.8	29.6	29.6	29.6
18-2	No BRAF mutation detected		29.9	30.7	29.6	29.8
18-3	No BRAF mutation detected		30.3	29.8	29.7	29.7

SUPPLEMENTAL FIGURES

Idylla amplification profiles of NGS-tested specimens with non-V600 *BRAF* variants that were ±5 amino acids of the BRAF V600 codon

Figure S1. *BRAF* Gly596Val
NGS result: *BRAF* c.1787G>T, p.Gly596Val (VAF: 6%),
HRAS c.34G>A, p.Gly12Ser (VAF: 12%)
Idylla software interpretation: NO MUTATION DETECTED IN *BRAF* CODON 600

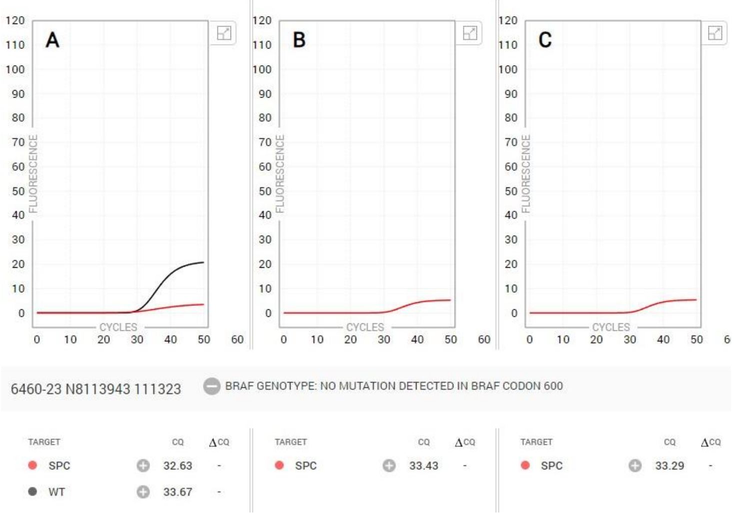

Figure S2. *BRAF* Leu597Arg
NGS result: *BRAF* c.1790T>G, p.L597R (VAF: 21%)
Idylla software interpretation: NO MUTATION DETECTED IN *BRAF* CODON 600

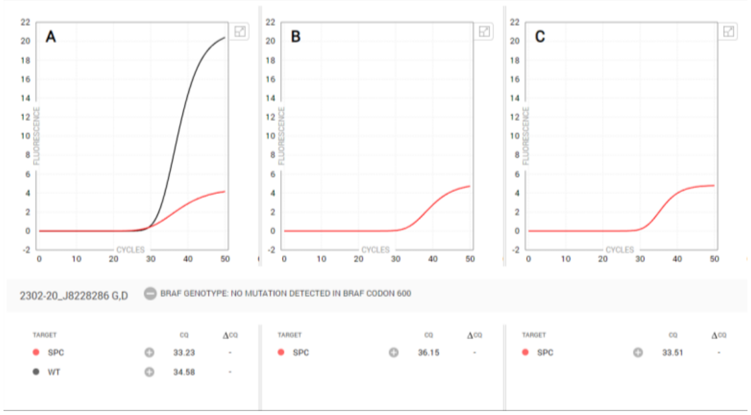

Figure S3. *BRAF* Leu597Val

NGS result: *BRAF* c.1789C>G, p.L597V (VAF: 42%)

Idylla software interp.: NO MUTATION DETECTED IN *BRAF* CODON 600

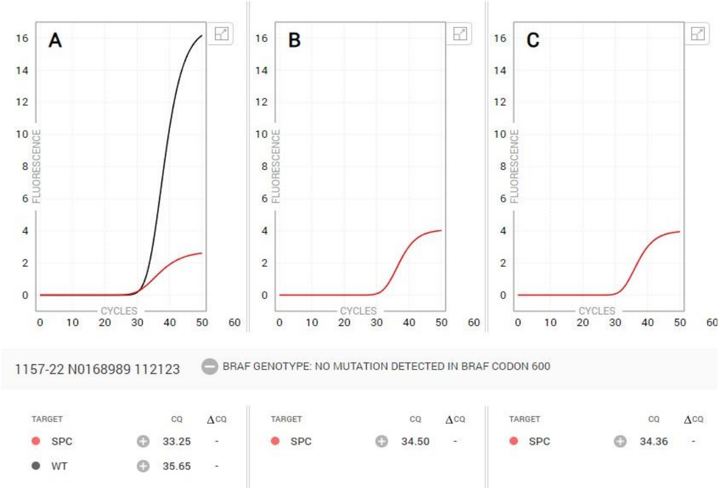

Figure S4. *BRAF* Leu597Gln

NGS result: *BRAF* c.1790T>A, p.L597Q (VAF: 13%);

KRAS c.38G>A, p.G13D (VAF: 14%),

ROS1 c.5827G>A, E1943K (VAF: 11%)

Idylla software interpretation: NO MUTATION DETECTED IN *BRAF* CODON 600

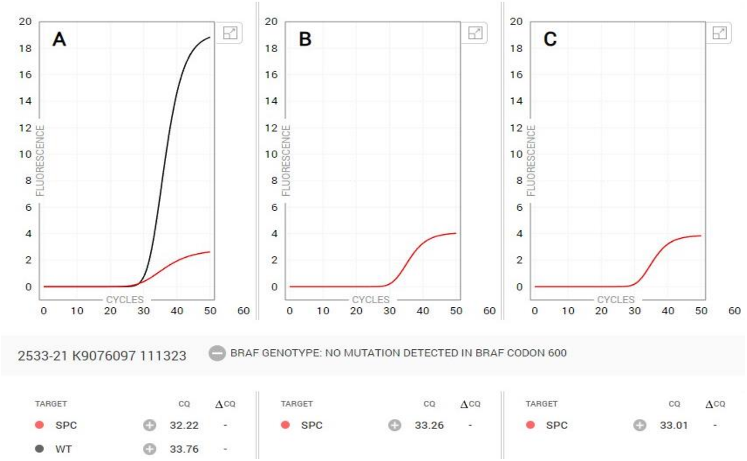

Figure S5. *BRAF* Ala598_Thr599insVal
NGS result: *BRAF* c.1794_1795insGTT, p.A598_T599insV
Idylla software interpretation: NO MUTATION DETECTED IN *BRAF* CODON 600

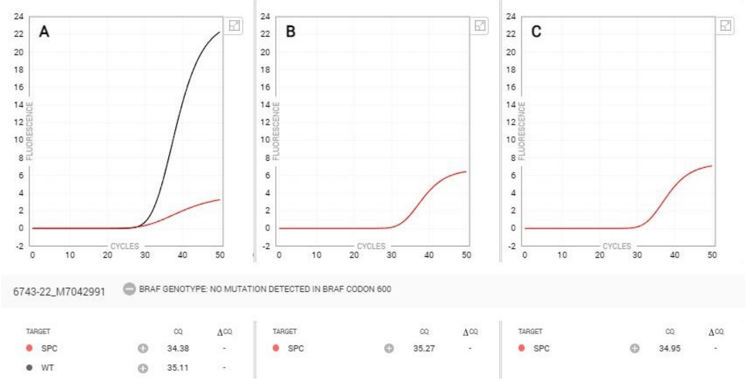

Figure S6. *BRAF* Lys601Glu
NGS result: *BRAF* c.1801A>G, p.K601E (VAF: 19%)
Idylla software interpretation: NO MUTATION DETECTED IN *BRAF* CODON 600

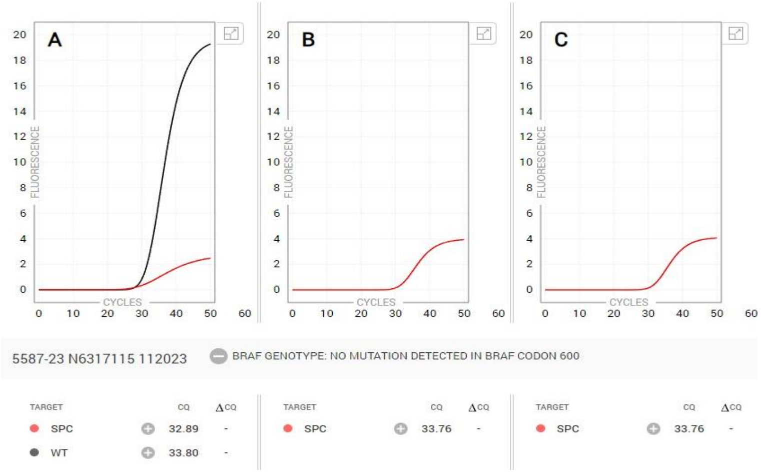

Figure S7. *BRAF* Lys601Thr

NGS result: *BRAF* c.1802A>C, p.K601T (VAF: 18%)

Idylla software interpretation: NO MUTATION DETECTED IN *BRAF* CODON 600

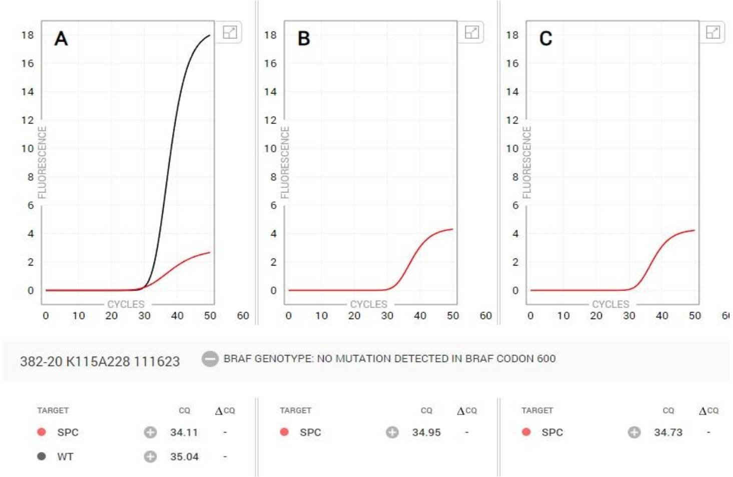

Figure S8. *BRAF* Lys601Asn

NGS result: *BRAF* c.1803A>C, p.K601N (VAF: 41%)

Idylla software interpretation: NO MUTATION DETECTED IN *BRAF* CODON 600

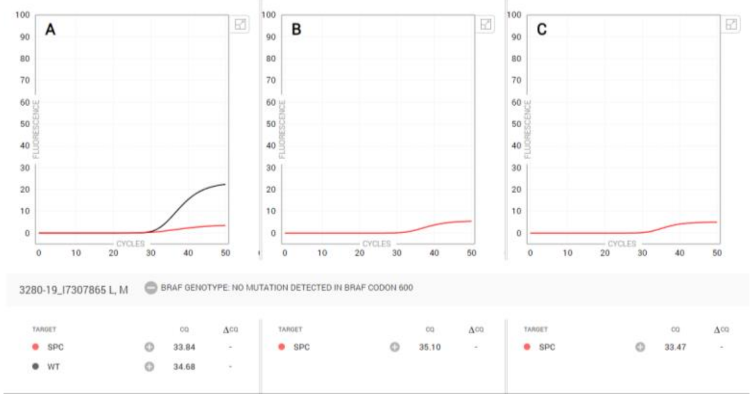

Figure S9. *BRAF* Trp604Cys

NGS result: *BRAF* c.1812G>C, p.W604C (VAF: 47%)
BRAF c.1780G>A, p.D594N (VAF: 47%),
KRAS c.34_35delGGinsTT, p.G12F (VAF: 49%)

Idylla result: NO MUTATION DETECTED IN *BRAF* CODON 600

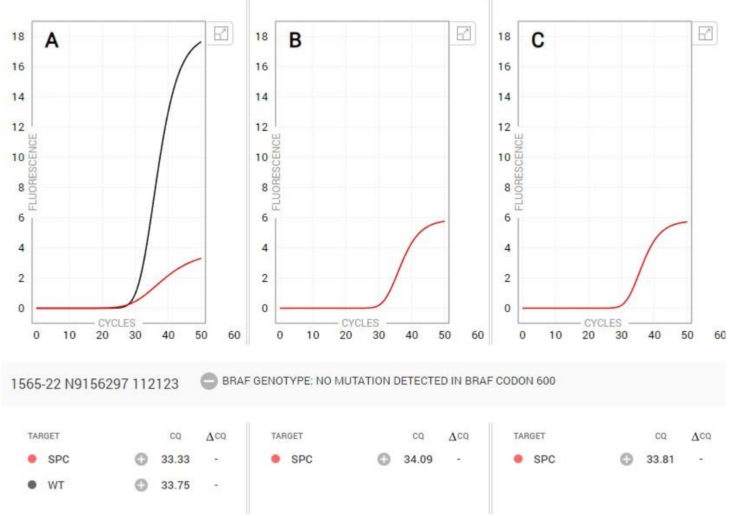

Supplement: Supplementary file 1 [file genes-15-00527-s001.zip › genes-2950391-supplementary.pdf]
